# Rosehip Extract Decreases Reactive Oxygen Species Production and Lipid Accumulation in Hypertrophic 3T3-L1 Adipocytes with the Modulation of Inflammatory State

**DOI:** 10.3390/nu16193269

**Published:** 2024-09-27

**Authors:** Katarzyna Kowalska, Anna Olejnik

**Affiliations:** Department of Biotechnology and Food Microbiology, Poznan University of Life Sciences, 48 Wojska Polskiego St., 60-627 Poznan, Poland; anna.olejnik@up.poznan.pl

**Keywords:** *Rosa canina*, hypertrophic adipocytes, oxidative stress, lipid accumulation, inflammation

## Abstract

Background: *Rosa canina* L. (rosehip) is used worldwide in traditional medicine as a plant with medicinal properties. However, its anti-obesity effects are not fully explained on a transcriptional level. Methods: In the present work, the 3T3-L preadipocytes were utilized to explore the impact of *R. canina* fruit extract (RCE) on the cellular and molecular pathways involved in adipocyte hypertrophy. Results: Obtained results showed the ability of RCE to reduce lipid overloads in hypertrophic adipocytes associated with the down-regulation of mRNA expressions of adipogenic transcription factors such as *PPARγ*, *C/EBPα*, and *SREBP-1c* as well as genes involved in lipid biosyntheses such as *FAS, LPL*, and *aP2*. Moreover, obesity-associated oxidative stress (antioxidant enzyme activities and ROS generation) and inflammation were ameliorated in RCE-treated hypertrophic adipocytes. The mRNA and protein levels of adipokines such as leptin, resistin, and adiponectin were restored to more favorable levels. Conclusions: *Rosa canina* fruit might be a valuable source of phytochemicals in preventing obesity and obesity-related metabolic complications.

## 1. Introduction

Excessive or abnormal fat aggregation in white adipose tissue (WAT) driven by the generation of new adipocytes in the adipogenesis process (hyperplasia) or by an increase in adipocyte size (hypertrophy) leads to obesity and accompanying metabolic disorders [1]. Adipose tissue hypertrophy changes the secretion of metabolites released and affects the surrounding microenvironment [2,3]. The hypertrophy of adipocytes increases the expression of pro-inflammatory mediators, such as TNF-α, IL-6, IL-1β, MCP-1, leptin, and resistin, in the adipose tissue. The release of pro-inflammatory adipokines into the bloodstream causes a systemic low-grade inflammation with a permanent increase in oxidative stress that enhances metabolic disorders such as insulin resistance, dyslipidemia, hypertension, and cardiovascular diseases (CVDs). Moreover, obesity leads to the accumulation of lipids in non-adipose organs, such as the liver, pancreas, muscle, epicardial, and perivascular tissue [2,4]. A deeper understanding of the pathologies of obesity on the cellular level is beneficial for obese patients in two aspects: preventing obesity and healing associated complications [2,4]. Dietary regimen are among the most important factors in preventing adiposity and associated disorders. Extensive research has shown that flavonoid intake is important in treating and preventing oxidative damage and chronic inflammation and exerts favorable biochemical and pharmacological effects on white adipose tissue hypertrophy [5]. Dietary flavonoids, which affect several molecular pathways that lead to adipocyte hypertrophy, can be a valuable and safe therapeutic strategy against adipogenesis and obesity [6].

*Rosa canina* L. is a member of the Rosaceae family with white flowers and red fruit, and its common name is “rosehip” [7]. The fruit of rosehip is used worldwide in traditional medicine [8,9,10]; it is known as a remedy for osteoarthritis, rheumatoid arthritis, cancer, kidney stones, depressive disorders, and dermatosis [8]. The high content of vitamins A, C, and E, and minerals (Ca, Mg, K, S, Si, Se, Mn, and Fe), along with other biologically active ingredients, makes *Rosa canina* fruit a beneficial source of functional compounds with antioxidant, anti-inflammatory, cardioprotective, antidiabetic, neuroprotective, and antimicrobial features [8]. Fruits are substantial sources of phytochemicals such as phenolic acids, flavones/ols, flavan3-ols, anthocyanins, flavonoids, carotenoids, fatty acids, and terpenoids [9]. Rosehip’s total phenolic content ranges between 290 and 1385 mg 100 g^−1^ [11], and the main phenolic compounds are catechins and procyanidin B2 [9]. Phenolic acids such as oleanolic, betulinic, citric, ursolic, cinnamic, caffeic, and ferulic acids were detected in *Rosa canina* fruit, and quercetin, rutin, and ellagic acid are usual flavonoids of the fruit [12,13]. Hydrolyzable tannins and galactolipids were also detected [12,13]. Moreover, rose hips have carotenoids such as chlorophyll and lycopene, and a high concentration of proanthocyanidins [8,9]. Unsaturated fatty acids, including linolenic and linoleic acids, and saturated fatty acids, such as palmitic and stearic acids, were found in rosehip seeds [13,14]. Studies report that rosehips have significantly higher vitamin C content than most fruits and vegetables [11]. Rosehips are rich in folate with a content ranging from 400 to 600 mg/100 g dry matter and 160 to 185 mg/100 g fresh weight [8,9].

The rich phytochemical composition of *Rosa canina* fruit makes it a potential source of natural products for treating and preventing obesity and associated metabolic pathologies. This study evaluated the effect of *Rosa canina* extract (RCE) on the cellular and molecular pathways involved in adipocyte hypertrophy using an in vitro 3T3-L1 model. We investigated the potential of the extract to diminish oxidative stress, inflammation, and dysregulated adipokine secretion in hypertrophic 3T3-L1 adipocytes. We investigated the potential of the extract to alleviate adipocyte hypertrophy via the modulation of oxidative stress and inflammation markers. Moreover, we examined the effect of RCE on the expression of adipokines and transcriptional factors involved in adipose tissue hypertrophy to elucidate the specific mechanisms by which *Rosa canina* fruit can affect health.

## 2. Materials and Methods

### 2.1. Preparation of Rosehip Extract

The rosehip (*Rosa canina* L.) was collected in the Natura 2000 region in September. They were freeze-dried, as previously described [15]. The freeze-dried rosehip was added to a culture medium and thoroughly mixed, then a solution pH was adjusted to 7.4. The extract was centrifuged (3000× *g*, 10 min) and the supernatant was sterilized by filtration using a 0.22 µm filter (Merck KGaA, Darmstadt, Germany). The hypertrophic adipocytes were treated with rosehip extract (RCE) on day 14 after differentiation with concentrations from 0.5 to 5 mg of rosehip powder/mL of complete medium.

### 2.2. Adipocyte Differentiation and Experiment Procedure

The mouse 3T3-L1 preadipocytes were acquired from the American Type Culture Collection (American Type Culture Collection, CL-173, Manassas, VA, USA). Preadipocytes were grown under controlled conditions at 37 °C and 5% CO_2_ atmosphere in a medium consisting of Dulbecco’s Modified Eagle’s Medium (DMEM) (Merck KGaA, Darmstadt, Germany) supplemented with 10% (*v*/*v*) calf serum (CS) (Thermo Fisher Scientific, Waltham, MA, USA). Preadipocytes were differentiated into adipocytes as described previously [16]. Cells were seeded onto 24-well plates with a 0.8 × 10^4^ cells/well density. Two days after confluency preadipocytes were cultured on a differentiation medium consisting of a complete culture medium with 0.25 µM DEX, 0.5 mM IBMX, and 1 µM insulin (Merck KGaA, Darmstadt, Germany). The medium was exchanged after 48 h with DMEM with 1 µM insulin. After another 48 h, the medium was replaced with the usual culture medium and exchanged every 48 h until adipocytes reached a hypertrophic state. RCE extract was added to the hypertrophic adipocytes for 24 h with concentrations ranging from 0.5 to 5 mg/mL.

### 2.3. Intracellular Reactive Oxygen Species Determination

Intracellular ROS production in hypertrophic adipocytes was examined by the nitroblue tetrazolium (NBT) assay as previously presented [17]. Cells were incubated for 90 min in 0.2% NBT solution (Merck KGaA, Darmstadt, Germany), rinsed with PBS, and fixed with methanol. Formazan was extracted from cells with 2M KOH and DMSO, and absorbance was read at 620 nm (Tecan Infinite M200, Tecan group Ltd., Männedorf, Switzerland).

### 2.4. Lipid Content Quantification

Lipid accumulation in hypertrophic adipocytes after RCE treatment was measured by the staining with Oil Red O dye (Merck KgaA, Darmstadt, Germany), as described formerly [15]. Cells were fixed for 1 h with 10% formalin, rinsed with 60% isopropanol, and thoroughly drained. Subsequently, the cells were incubated for 15 min with Oil Red O working solution and rinsed thoroughly four times with water. The images of stained cells were acquired using an inverted contrast phase microscope (Axiovert 40 C, ZEISS AG, Oberkochen, Germany). After extraction of red-stained lipids with isopropanol, the absorbance was read at 500 nm.

### 2.5. Quantitative Real-Time PCR Analysis

TRI-Reagent (Merck KGaA, Darmstadt, Germany) was used for total RNA extraction from cells. The first-strand cDNA synthesis was carried out with a Transcriptor High Fidelity cDNA Synthesis Kit (Merck KGaA, Darmstadt, Germany). Gene expression was quantified using a CFX96 Real-Time System with a C1000 TouchTM Thermal Cycler (Bio-Rad, Hercules, CA, USA). The sequences of specific primers used for gene expression analysis are noted in Table S1. The PCR reaction consisted of 1 µL cDNA, 1 µL of specific forward and reverse primers (5 µM), 12.5 µL SYBR^®^ Select Master Mix (Thermo Fisher Scientific, Waltham, MA, USA), and nuclease-free water to a final volume of 25 μL. The PCR reaction mix was denatured at 94 °C for 10 min, followed by 40 cycles of 95 °C for 40 s, 59 °C for 30 s, and 72 °C for 30 s. The 2^−∆∆CT^ method was applied to calculate the relative mRNA levels of individual genes. The levels of each transcript were standardized to β-actin. The gene expression results are presented as fold change compared to the control (untreated) cells. All analyses were performed in triplicate.

### 2.6. Quantification of Adipokines

The leptin, resistin, and adiponectin levels were measured using ELISA kits (Merck KGaA, Darmstadt, Germany). Quantitation was carried out by calibration with standards. The concentration of adipokines is presented in ng/mL of culture medium, corresponding to the protein quantity per 1 × 10^6^ cells. Standards, control, and samples were measured in triplicate.

### 2.7. Statistical Data Analysis

The STATISTICA version 13.3 software (Statsoft, Inc., Tulsa, OK, USA) was used for statistical data analysis. The difference between groups’ mean values was calculated by the one-way analysis of variance (ANOVA) and Tukey’s post hoc test. Levene’s test confirmed the assumption of equality of variation. A *p*-value of <0.05 was set as statistically important. Data are presented as the means ± SD from three independent analyses.

## 3. Results and Discussion

### 3.1. The Effect of Rosehip Extract on Lipid Accumulation and Lipogenic Genes Expression

Large, hypertrophic fat cells are distinguished as less metabolically favorable and are linked with pathological conditions. Dysfunctional lipid-overloaded adipocytes have impaired cellular function and lose the ability to maintain lipid homeostasis. Therefore, reducing large fat cells is essential for improving adipose tissue function and health. There is a strong connection between adipose cell size and cellular function, and adipocyte size is a significant factor that contributes to the progress of pathological conditions [18].

Hyperplasia occurs through adipogenesis under the control of several transcription factors such as peroxisome proliferator-activated receptor γ (PPARγ), CCAAT/enhancer-binding protein α (C/EBPα), and lipogenic transcription factor sterol regulatory element-binding protein 1c (SREBP-1c). PPARγ induces the expression of other target genes such as C/EBPα, adipocyte protein 2 (aP2), lipoprotein lipase (LPL), acyl-CoA synthase, and fatty acid synthase (FAS) [18]. SREBP-1c is a key regulator of the expression of FAS and LPL, genes participating in fatty acid metabolism [19]. FAS is mainly expressed in adipose tissue and liver and has been recognized as a therapeutic target for obesity. In obese mice, FAS inhibitors were shown to reduce food intake and body weight [20]. In 3T3-L1 adipocytes after 14 days of differentiation, increased mRNA expression of PPARγ, C/EBPα, LPL, PLIN1, aP2, and leptin was observed [21]. 

**Figure 1:**
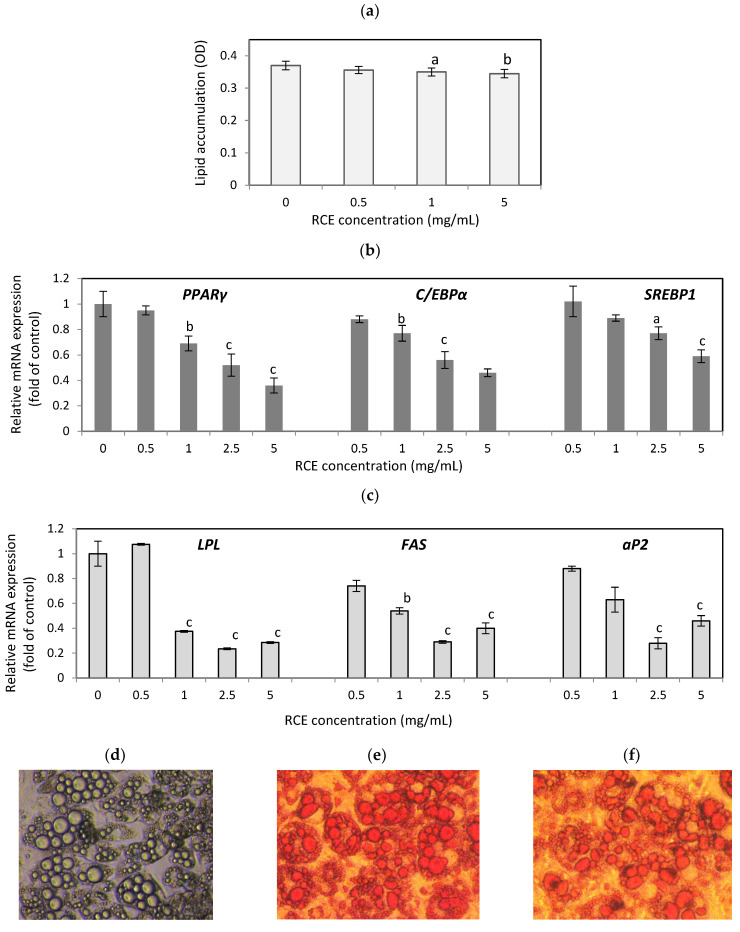

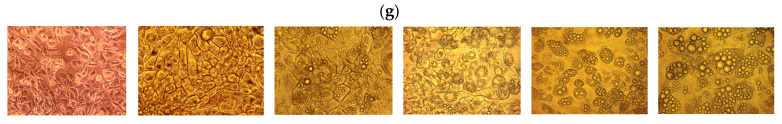
Effect of RCE on lipid amounts after Oil Red O staining (**a**) and lipogenic genes’ expression (**b**,**c**) in hypertrophic 3T3-L1 adipocytes. Data are the mean values ± SD (*n* = 3). ^a^ *p* < 0.05, ^b^ *p* < 0.01, ^c^ *p* < 0.001. The images show the hypertrophic adipocytes after 14 days of differentiation (**d**), Oil Red-stained adipocytes non-treated (**e**)**,** and treated with 5 mg/mL RCE (**f**). The images in (**g**) show the different steps of the differentiation process, from preadipocytes to hypertrophic adipocytes. Adipocytes were photographed at a magnification of 100×.

To study the effect of *Rosa canina* on lipid accumulation, hypertrophic adipocytes were treated on day 14 for 24 h with RCE extract, and the amount of lipids in the cells was quantified by Oil Red O staining. The lipids accumulation was reduced by 3.8%, 5.4% (*p* < 0.05), and 6.8% (*p* < 0.01) at a concentration of 0.5, 1, and 5 mg/mL, respectively (Figure 1a). Its influence on the expression of adipogenic genes was examined to investigate the mechanism by which RCE inhibits lipid accumulation. Real-time PCR analysis showed that RCE significantly decreased the expressions of adipogenic transcription factors *PPARγ, C/EBPα*, and *SREBP-1c*, as well as their target genes (*FAS*, *aP2*, and *LPL*) (Figure 1b,c). RCE at a concentration of 1, 2.5, and 5 mg/mL decreased mRNA levels of *PPARγ* by 31%, 48%, and 64% (*p* < 0.001), *C/EBPα* by 23%, 44%, and 54% (*p* < 0.001), and *SREBP-1* by 11%, 23%, and 41% (*p* < 0.001), respectively. The mRNA level of *FAS*, *LPL*, and *aP2*, involved in lipid metabolism, was significantly lower after RCE treatment. Compared to untreated cells, *LPL* expression decreased by 62%, 76%, and 71% (*p* < 0.001), *FAS* decreased by 46%, 71%, and 60% (*p* < 0.001), and *aP2* decreased by 37%, 72%, and 54% (*p* < 0.001) (at an RCE concentration of 1, 2.5, and 5 mg/mL).

Nagatomo et al. (2013) have shown that rosehip extract (RHE) and tiliroside, the principal constituent of its seeds, inhibited lipid accumulation in a dose-dependent manner in differentiated 3T3-L1 cells, as well as reduced lipid accumulation in WAT of obese mice. Compared to the control group, mice on a diet containing 1% RHE had less visceral fat and gained less body weight. A substantial reduction in the expression of PPARγ was noticed in epididymal fat in the RHE-fed mice, suggesting that down-regulation of PPARγ expression might be, at least partially, involved in the suppressive effect of RHE on lipid accumulation in WAT [22]. Another study has shown the preventive effect of *R. canina* on high-fat diet (HFD)-induced obesity. Mice on HFD supplemented with *R. canina* for 18 weeks gained less body weight and showed reduced hyperglycemia and insulin resistance. The white adipose tissue of *R. canina*-supplemented mice showed a larger amount of smaller adipocytes, with decreased lipogenesis and a significantly reduced expression of genes related to lipogenesis, fatty acid oxidation, and glucose metabolism [23]. In small clinical trials, intake of rosehip extracts or powder reduced abdominal adiposity and cardiovascular risk factors associated with obesity [24,25]. Many studies have shown a preventive effect of flavonoids on obesity both in vitro and in vivo, emphasizing that combining many phytochemicals rather than single compounds had a strong synergistic effect on inhibiting lipid accumulation [26]. The synergistic effect of flavonoids may be beneficial in treating diseases related to lipid accumulation.

### 3.2. The Effect of Rosehip Extract on Oxidative Stress

In pathological conditions, adipose tissue induces reactive oxygen species (ROS) production, generating oxidative stress (OS) that leads to irregular adipokine production and the release of pro-inflammatory cytokines. OS and pro-inflammatory processes are strongly related, and ROS synthesis promotes an inflammatory status in adipose tissue [27]. Because oxidative stress in obesity is a critical component in developing obesity-related complications, factors with high antioxidant properties, such as natural bioactive compounds, are beneficial to obese people, decreasing ROS generation and lowering inflammatory state.

**Figure 2:**
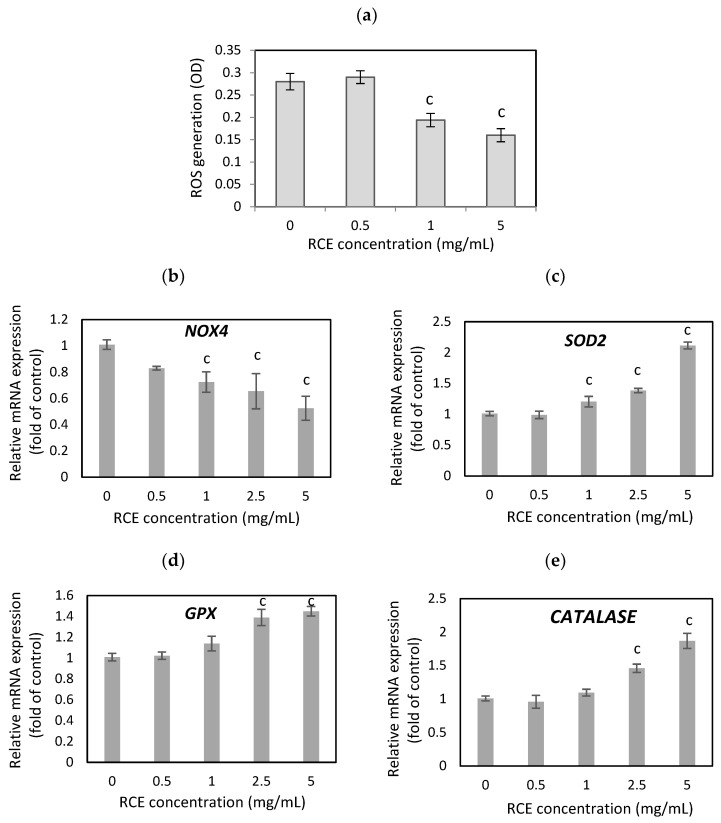
Effect of rosehip extract (RCE) on the ROS production after NBT staining procedure (**a**), and pro-oxidant (**b**) and antioxidant enzymes’ (**c**–**e**) mRNA expression in hypertrophic adipocytes. Data are the mean values ± SD (*n* = 3). ^c^ *p* < 0.001.

The results from the NBT assay have shown that treatment of the hypertrophic 3T3-L1 cells with RCE extract for 24 h remarkably diminished the elevation of ROS levels in adipocytes (Figure 2a) with no effect on adipocyte viability. The RCE extract at 1 and 5 mg/mL concentrations decreased the ROS generation by 30.7% and 42.9% (*p* < 0.001) compared to untreated adipocytes. Moreover, evidence indicates that ROS overproduction decreases the efficiency of antioxidant defenses. The lower levels of antioxidant enzymes and increased NADPH oxidase 4 (NOX4) activity were observed in obese patients [27]. NOX, especially NOX4, is a primary source of ROS synthesis in adipocytes and plays an essential part in adipogenesis. Overexpression of NOX4 in human preadipocytes enhanced the accumulation of fat droplets, while NOX4 knockdown inhibited adipocyte differentiation [28]. RCE extract significantly down-regulated *NOX4* expression in hypertrophied adipocytes by 27.5%, 34.5%, and 47.5% at concentrations of 1, 2.5, and 5 mg/mL, respectively (*p* < 0.001) (Figure 2b). Hua et al. (2023) have found that reducing NOX4 activity in the adipose tissue of obese mice contributes to the anti-obesity effect by inhibiting adipocyte differentiation and lipid accumulation and improving glucose metabolism [29].

The first-line defense antioxidant enzymes, which include superoxide dismutase (SOD), catalase (CAT), and glutathione peroxidase (GPX), play a fundamental part in preventing oxidative stress and the attendant cellular damage [30]. Dysregulated GPX expression is associated with severe pathologies, which include obesity and diabetes [31]. We found that RCE extracts significantly reduced the expression of *NOX4* and remarkably enhanced the expression of antioxidant defense enzymes. The expression of *SOD2, GPX*, and *CAT* (Figure 2c,d) in hypertrophied adipocytes after RCE treatment (1, 2.5, and 5 mg/mL) increased by 20.5%, 38.5%, and 111.5% (*p* < 0.001) for *SOD2*, by 14%, 39%, and 45% (*p* < 0.001) for *GPX*, and by 9.7%, 46%, and 87% (*p* < 0.001) for *CAT*. Forouzanfar et al. (2023) showed that *Rosa canina* fruit extract reduced ROS production in HUVEC cells induced by H_2_O_2_ treatment [32]. The study conducted by Sadeghi et al. (2016) demonstrated the hepatoprotective effects of the ethanolic extract of *R. canina* fruit on CCl4-induced hepatic damage in rats. These protective effects could be, at least partially, related to the antioxidant properties of the extract [33]. Another study showed that oral *Rosa canina* administration reduced rats’ nephrotoxicity, possibly through antioxidant pathways [34]. Administration of *Rosa canina* in rats significantly improved cognitive dysfunction induced by heat stress, reduced reactive oxygen species levels, and enhanced antioxidant defense in the hippocampus [35]. High vitamin C concentration and the phenolic compounds in *R. canina* fruit may be responsible for this beneficial effect [36,37]. It has been shown that flavonoids, naturally occurring antioxidant compounds, affect the regulation of lipid metabolism by modulating oxidative stress conditions [38]. Intake of antioxidant-rich natural products might enhance the body’s potential to reduce oxidative stress-related health complications.

### 3.3. The Effect of Rosehip Extract on Adipokines Expression and Secretion

Hypertrophic adipocytes have an altered hormone secretion profile compared with lean adipocytes. Lipid-overload adipocytes overproduce leptin, causing hyperleptinemia and leptin resistance [39]. A study conducted by Zelissen et al. (2005) has shown that the reduction of leptin levels by genetic modulation reduced weight gain and improved insulin sensitivity in obese mice, demonstrating that a precise leptin-lowering therapy may benefit obese individuals [40]. In animals, reduced adiponectin production in hypertrophic adipocytes was linked with insulin resistance and poor metabolic health, whereas animals overexpressing adiponectin were protected against the inflammatory and metabolic effects of severe obesity [39,41]. It has been demonstrated that increased levels of circulating resistin in obese animals induce insulin resistance, and reduced resistin gene expression improves insulin sensitivity and glucose homeostasis [42,43]. There is a correlation between high resistin serum levels and human atherosclerosis [44].

**Figure 3:**
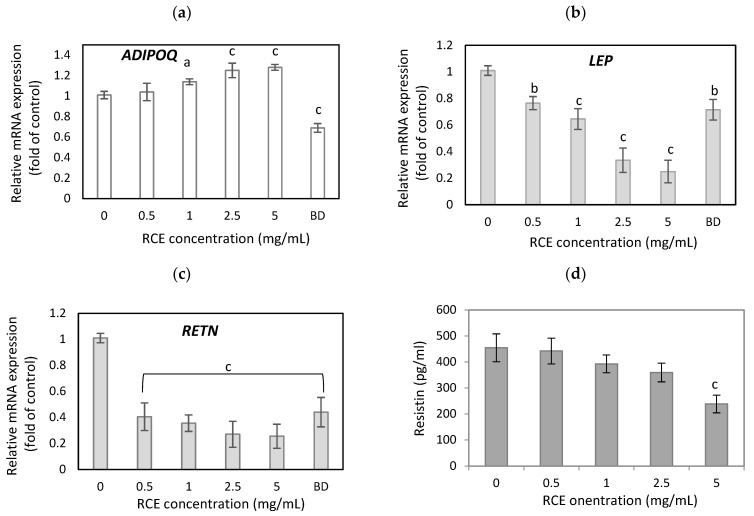

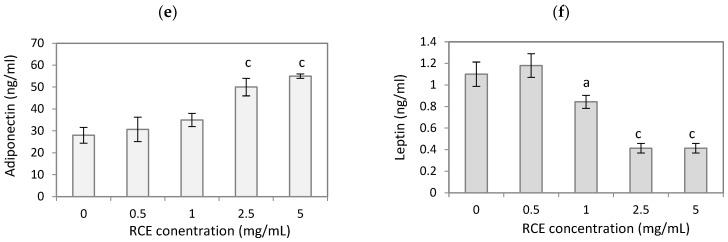
The effect of RCE on adiponectin (**a**), leptin (**b**), and resistin (**c**) gene expression and adiponectin (**e**), leptin (**f**), and resistin (**d**) protein secretion by hypertrophic adipocytes. Budesonide (BD) was used as a positive control. Data are the mean values ± SD (*n* = 3). ^a^ *p* < 0.05, ^b^ *p* < 0.01, ^c^ *p* < 0.001.

In our study, leptin and resistin gene expression significantly decreased after RCE treatment (Figure 3b,c). Leptin mRNA levels were reduced by 23.5%, 35.5%, 66.5%, and 75% (*p* < 0.001) at an RCE concentration of 0.5, 1, 2.5, and 5 mg/mL compared to untreated cells (Figure 3b). As a positive control, we used budesonide, a potent topical anti-inflammatory agent, which inhibited leptin gene expression by 28.5%. (*p* < 0.01). RCE extract had a more powerful effect on resistin level, decreasing mRNA expression by 59.5%, 64.5%, 73%, and 74.5% (*p* < 0.001) with increasing concentrations of RCE, while budesonide inhibited resistin level by 56% *(p* < 0.001). RCE also positively affected adiponectin gene expression (Figure 3a). RCE at a concentration of 1, 2.5, and 5 mg/mL enhanced adiponectin mRNA levels in hypertrophic adipocytes by 14%, 25%, and 28% (*p* < 0.001) compared to untreated cells. A similar correlation was observed in the protein levels of adipokines after RCE treatment (Figure 3d–f).

A meta-analysis of randomized controlled trials (40 publications) exploring the effects of flavonoid intake on leptin and adiponectin levels demonstrated that flavonoid intervention significantly decreased leptin levels and elevated adiponectin level [45]. The supplementation of diabetic animals with flavonoids markedly ameliorated the depressed adipose tissue adiponectin mRNA expression and decreased the elevated IL-6 mRNA expression [46]. Mohri et al. (2022) identified lycopene and β-carotene as food-derived metabolites that activate the adiponectin signaling pathway [47]. In a randomized, double-blind, placebo-controlled trial, overweight or obese women on quercetin supplementation over 12 weeks had a remarkably lower resistin concentration and mRNA level compared with the placebo group [48].

Taken together, leptin, resistin, and adiponectin levels may be biomarkers of inflammatory and metabolic disease, and therapies that target their levels could stop or limit the obesity epidemic. Thus, *Rosa canina* fruit supplementation might be an efficient strategy for improving the adiponectin, leptin, and resistin ratio and, consequently, may benefit obese people with accompanying metabolic disorders.

### 3.4. The Effect of Rosehip Extract on Inflammatory Cytokine Expression

Compared to small fat cells, large, hypertrophic adipocytes are highly inflammatory and release significant amounts of potent inflammatory cytokines, such as tumor necrosis factor-alpha (TNFα), interleukin-6 (IL-6), interleukin-1β, and monocyte chemoattractant protein-1 (MCP-1); thus, obesity can be considered as an inflammatory immune disease [39,49,50]. Many different cells and tissues produce and secrete IL-6. However, white adipose tissue provides as much as 35% of circulating IL-6, and weight reduction was parallel with decreased mRNA and protein levels of IL-6 in subcutaneous adipose tissue of human subjects [51]. In mice, increased expression of MCP-1 in adipose tissue enhances macrophage infiltration, insulin resistance, and hepatic steatosis [52]. People with sarcopenic obesity show detrimental changes in myokines and adipokines secretion associated with decreased levels of interleukin-10 (IL-10), insulin-like growth factor hormone (IGF-1), fibroblast growth factor-21 (FGF-21), and adiponectin, while myostatin, leptin, IL-6, and resistin increase. These changes elevate the inflammatory status, increasing fat mass and decreasing muscle tissue, thus exacerbating sarcopenia obesity [53]. It has been shown that childhood obesity with hypertriglyceridemia leads to an inflammatory state connected with a low IL-10 and adiponectin expression in adipose tissue and a higher leptin-to-adiponectin ratio. Furthermore, the IL-10 expression was negatively correlated with triglyceride and LDL-C levels [54]

**Figure 4:**
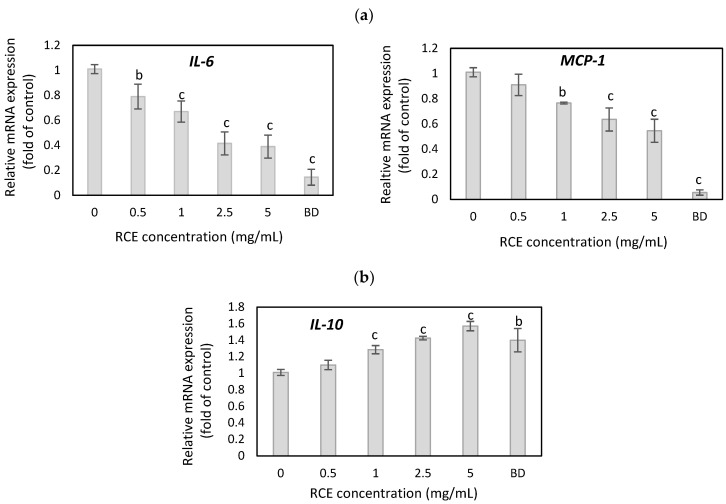
The effect of RCE on pro-inflammatory IL-6 and MCP-1 (**a**) and anti-inflammatory IL-10 (**b**) gene expression in hypertrophic adipocytes. Budesonide (BD) was used as a positive control. Data are the mean values ± SD (*n* = 3). ^b^ *p* < 0.01, ^c^ *p* < 0.001.

The effects of *Rosa canina* extracts on inflammatory status in hypertrophic adipocytes remain poorly investigated. In this study, the anti-inflammatory capability of *Rosa canina* fruit was examined in hypertrophic adipocytes by analyzing the expression of pro-inflammatory genes such as *IL-6* and *MCP-1* and an anti-inflammatory *IL-10* expression. As shown in Figure 4a, RCE extract at a concentration of 1, 2.5, and 5 mg/mL decreased significantly mRNA level of *IL-6* by 33%, 58.5%, and 61% (*p* < 0.001) and *MCP-1* mRNA level by 23.5%, 36.5%, and 45.5% (*p* < 0.001) when compared to untreated adipocytes. Budesonide, a potent anti-inflammatory agent, inhibited *IL-6* expression by 85.5% and *MCP-1* by 94.5% (*p* < 0.001), respectively. Real-time PCR analysis showed that *IL-10* mRNA in hypertrophic adipocytes after RCE treatment increased by 28.5%, 42.5%, and 57% (*p* < 0.001) (at 1, 2.5, and 5 mg/mL) when compared to untreated cells (Figure 4b). Budesonide increased the *IL-10* level by 40% (*p* < 0.01) compared to the control.

Potent natural antioxidants have been shown to improve inflammatory responses and energy metabolism due to decreasing the levels of inflammatory adipokines, including IL-6, MCP-1, leptin, and TNFα, thus attenuating obesity-associated complications [55,56]. Flavonoids, such as rutin and kaempferol, reduced TNF-α and IL-1β expression and lipid peroxidation and enhanced antioxidant defense, contributing to reduced body weight gain in diabetic animals [56]. Luteolin and tangeretin decreased the levels of circulating inflammatory cytokines such as IL-6, MCP-1, leptin, and resistin [56]. In inflamed macrophages, rosehip powder inhibited PGE2 and NO production and inhibited the release of cytokines (TNF-α, IFN-g, IL-1β, IL-6, and IL-12) and chemokines [57]. In LPS/IFN-γ-activated human peripheral blood leukocytes, rosehip powder diminished the secretion of chemokines and cytokines, including IL-6 and IL-12. Most effects were on the transcriptional level, since rosehip powder significantly influenced gene expression [58].

## 4. Conclusions

In summary, these results have shown that *Rosa canina* fruit ameliorates adipocyte hypertrophy by influencing the molecular and cellular pathways involved in lipid metabolism, oxidative stress, and inflammatory processes. Most effects were observed on the transcriptional level. RCE extract enhanced the expression of antioxidant defense enzymes such as *SOD2*, *GPX*, and *CAT*, and inhibited oxidant *NOX4*, thus decreasing intracellular ROS generation and reducing oxidative stress in hypertrophic adipocytes. Moreover, RCE, by down-regulating the expression of *PPARγ*, *C/EBPα*, and *SREBP-1* and their target genes, such as *LPL*, *FAS*, and *aP2*, reduced adipocyte lipid content. The elevated mRNA level of inflammatory factors (*IL-6* and MCP-1) and adipokines such as leptin and resistin were down-regulated, and anti-inflammatory *IL-10* and adiponectin expression was up-regulated in hypertrophic adipocytes after RCE treatment.

These results indicated the potential applications of *Rosa canina* fruit in treating and preventing the pathological function of obese adipocytes. Furthermore, the diverse rosehip bioactivities make it a valuable ingredient for developing functional foods, nutraceuticals, and pharmaceuticals that can contribute to health and well-being. The targeted intervention on lipid metabolism, oxidative stress, and inflammatory pathways may help to prevent the dysfunctional effect of adipose tissue hypertrophy. Hypertrophic, lipid-overloaded adipocytes have impaired adipokine and cytokine secretion, which leads to inflammation and oxidative stress in adipose tissue. Therefore, preventing or treating adipocyte hypertrophy may be one of the most important strategies in the fight against obesity and concomitant diseases.

## Data Availability

Research data can be found at https://doi.org/10.18150/FSS05Y.

## Supplementary Materials

The supporting information can be downloaded at https://www.mdpi.com/article/10.3390/nu16193269/s1, Table S1: The primers sequence used for real-time PCR.
