# Supplementary material for: Rosehip Extract Decreases Reactive Oxygen Species Production and Lipid Accumulation in Hypertrophic 3T3-L1 Adipocytes with the Modulation of Inflammatory State
Source: Nutrients. 2024 Sep 27;16(19):3269. doi: 10.3390/nu16193269 (PMC11478984; doi:10.3390/nu16193269)
Supplement: Supplementary file 1 [file nutrients-16-03269-s001.zip › nutrients-3175727-supplementary.pdf]

Table S1. The primers sequence used for real-time PCR

Gene	Accession	No. Sequence (5' – 3')	Amplicon (bp)
**Mm PPARγ**	NM-011146	F: TTTTCAAGGGTGCCAGTTTC<br>R: AATCCTTGGCCCTCTGAGAT	198
**Mm *C/EBPα***	NM-007678	F: TTACAACAGGCCAGGTTTCC<br>R: GGCTGGCGACATACAGTACA	188
**Mm *SREBP1***	NM-011480	F: TGTTGGCATCCTGCTATCTG<br>R: AGGGAAAGCTTTGGGGTCTA	190
**Mm *FAS***	NM-007988	F:TTGCTGGCACTACAGAATGC<br>R:AACAGCCTCAGAGCGACAAT	192
**Mm *LPL***	NM-008509	F: TCCAAGGAAGCCTTTGAGAA<br>R:CCATCC TCAGTCCCAGAAAA	188
**Mm *aP2***	NM-024406	F: TCACCTGGAAGACAGCTCCT<br>R: AATCCCCATTTACGCTGATG	182
**Mm *MCP-1***	NM-011333.3	F:TTCCTCCACCACCATGCAG<br>R:CCAGCCGGCAACTGTGA	64
**Mm *IL-6***	NM-031168.1	F:TCTGAAGGACTCTGGCTTTG<br>R:GATGGATGCTACCAAACTGGA	142
**Mm *IL-10***	NM-010548.2	F:CAGGGATCTTAGCTAACGGAAAC<br>R:GCTCAGTGAATAAATAGAATGGGAAC	110
**Mm *NOX4***	NM-015760.5	F:GATCACAGAAGGTCCCTAGCAG<br>R:GTTGAGGGCATTCACCAAGT	134
**Mm *SOD2***	NM-013671.3	F:CGTGTCTGTGGGAGTCCAAGGTTCAG<br>R:GTCAATCCCCAGCAGCGGAATAAG	139
**Mm *GPX***	NM-008160.6	F:GGGCAAGGTGCTGCTCATTG<br>R:AGAGCGGGTGAGCCTTCTCA	269
**Mm *CAT***	NM-009804.2	F:CCTCCTCGTTCAGGATGTGGTT<br>R:CGAGGGTCACGAACTGTGTCAG	243
**Mm *LEP***	NM-008493	F:GGA TCA GGT TTT GTG GTG CT<br>R:TTG TGG CCC ATA AAG TCC TC	187
**Mm *ADIPOQ***	NM-009605	F:CTGGCCACTTTCTCCTCATTTC<br>R:GGCATGACTGGGCAGGATTA	120
**Mm *RESTN***	NM-0022984.4	F: TCATTTCCCCTCCTTTTCCTTT<br>R: TGGGACACAGTGGCATGCT	70
ACTB	NM-007393	F:CCA CAG CTG AGA GGG AAA TC<br>R:AAG GAA GGC TGG AAA AGA GC	193
